# London Street Accidents

**Published:** 1913-05-17

**Authors:** 


### The Hospital

The Workers' Newspaper of Administrative Medicine and Institutional Life, Administration, National Insurance and Health.

No. 1402, Vol. LIV. SATURDAY, MAY 17, 1913. PRICE ONE PENNY.

### LONDON STREET ACCIDENTS.

The inquiry which has been held lately into the subject of traffic control and street accidents in London has been productive of widely differing opinions and curiously divergent interpretations of statistics. Lord Montagu of Beaulieu, for instance, offered some very instructive comments tending to support the thesis that accidents due to mechanically driven vehicles are no more frequent in proportion than such accidents used to be. Lord Montagu, as is well known, is an enthusiastic motorist and a keen supporter of aerial locomotion. He has, moreover, always exerted his great influence to check reckless driving and carelessness of the interests of pedestrians on the roads. His evidence certainly proved that statistics about traffic dangers need very careful sifting, and are capable of misleading those who merely glance at them in a cursory way. But, granting all that, the fact remains, and cannot be explained away, that fatal accidents due to motor traffic in our streets are increasing in number at a rate which constitutes a very serious item in our bills of mortality.

Let it be quite clearly understood that the enormous increase of motor traction was certain to produce and to account for an excess of fatal (and other) street accidents in every successive year. At least it would have been strange, though gratifying, had it been otherwise. Let us admit also that if the petrol engine had never been invented, the total number of fatalities by traffic accidents in London would probably have increased, more or less regularly, year by year. Still, the figures show conclusively that motors have been responsible for a large number of deaths which would certainly not have occurred with horse traction alone.

The figures of the Metropolitan Police District prove this to demonstration. In 1905 the total number of licensed horse-drawn public conveyances (cabs, omnibuses, trams) was 15,201: they caused twenty-four fatal accidents. In 1912 the number licensed had decreased to 4,223; the deaths were six, practically the same in proportion as before. When we turn to motor vehicles the figures tell quite another tale. Of these there were in 1905 4,627 licensed; and they accounted for sixteen deaths. In 1912 there were 13,736 licensed, and 244 deaths are ascribable to them. It may be true—indeed it is morally certain—that such vehicles accomplish, on the average, a far greater annual mileage than they did eight years ago; but even allowing for that, it is impossible to get away from the plain facts of this appalling wastage of human life in the Metropolis and in other large cities of the United Kingdom. Let us examine the statistics in another way. In 1905 the total reported fatalities from horse-drawn vehicles of all kinds, licensed and private, was 118; in 1912 this had risen to 145. Motor traction caused in 1905 but thirty-five deaths, whereas in 1912 the number was 369. Thus, the total number of these catastrophes was more than three times as great in 1912 as it was in 1905; and the vast part of this increase is traceable to motor traction.

What then is the remedy? To begin with, it is quite futile to attempt any artificial restriction of motor transport. It has come to stay, or at least to stay until some more economical or more convenient method of converting potential energy into momentum can be devised by the ingenuity of engineers. Speed limits, again, are an expedient to be applied with discretion, for they are only valuable in certain kinds of streets, and even at their best they do not nearly abolish the possibility of accidents. This the police are well aware of, and it has been through the expert advice of the heads of the Force that several applications for speed limits in various parts of London have been refused by the Local Government Board. Greater care in driving would unquestionably conduce to fewer accidents and fewer deaths; and any steps which will impress this upon motor drivers should command attention. It is true that the general level of driving is extremely high, and that the majority of drivers are as careful and conscientious as it is possible to wish. It is also true that in many of the accidents which do occur there is contributory negligence, often of a gross order, on the part of the pedestrian concerned. But all the same, there does exist a certain type of driver whose method is fraught with the risk of accident; and in the eradication of this faulty driving lies the best hope of reducing street accidents to a minimum. It is not that speeds excessive in themselves are wantonly practised: to drive down Cromwell Road or Portland Place at twenty miles an hour is perfectly safe in theory. Nor is there any laxity about endeavours to avoid accident when one appears imminent. Where the common fault lies is in failure to take such steps as shall suffice to avert accident *supposing some unexpected emergency arises suddenly.* To slow up for sharp corners and blow a horn as they are approached is an elementary precaution of this sort which any decent driver takes. The emergency which should be always foreseen, and frequently is not, is the sort which occurs by reason of the carelessness of foot passengers themselves or of the drivers of other vehicles. The motor driver who has a margin in hand sufficient to cover these risks may not reach his destination quite so quickly as his rivals; but he will keep a clean licence, and as drivers in the mass approach nearer and nearer to this ideal, so will the police returns of fatal accidents diminish instead of increasing. How to bring this about may be left to motor experts to suggest; but it seems to us certain that no other solution of the problem is half so likely to remove this grave reproach from modern London.

